# Xenobiotic Hazards in Aircraft Cabin Air

**DOI:** 10.3390/jox16040119

**Published:** 2026-06-23

**Authors:** Jeremy J. Ramsden

**Affiliations:** 1 Centre for Molecular Recognition, Collegium Basilea (Institute of Advanced Study), 4053 Basel, Switzerland; j.ramsden@colbas.org; 2 Department of Biomedical Research, Faculty of Medicine and Health Sciences, The University of Buckingham, Buckingham MK18 1EG, UK; 3 Department of Materials, School of Applied Sciences, Cranfield University, Bedfordshire MK43 0AL, UK

**Keywords:** cholinesterase, J-value, nanoparticles, organophosphates, triaryl phosphate, tributyl phosphate, tritolyl phosphate

## Abstract

Most airline passengers and crew assume that the air in the cabin is free from harmful or hazardous substances, as is mandated by airworthiness regulations. While fresh air entering the cabin is sterile (and if recirculated is usually efficiently filtered to remove microorganisms), if the fresh air is bled off the turbine compressors (as is the case in about 95% of airliners currently in service), it may be contaminated with traces of engine oil and ultrafine particles abraded from the turbine blades, and possibly traces of hydraulic fluid leaking from servo systems. Engine oil contains tricresyl phosphate (TCP) as an essential antiwear agent, but it is also a well-known neurotoxin, and it has been suggested that there may be no safe lower limit of exposure, not least because of considerable variation among individuals in sensitivity to tri-*ortho*-cresyl phosphate (T*o*CP) and other isomers with at least one *ortho* constituent. This paper reviews current knowledge about these hazards and discusses the medical and economic motivations for diminishing them. A calculation based on maintaining the life quality index shows that eliminating xenobiotic hazards in aircraft cabin air is likely to be affordable.

## 1. Introduction—The Need to Pressurize Jetliner Cabins and Biotic Hazards

Flying is an unnatural activity for human beings, and its realization can be expected to result in exposure to unnatural hazards. Many of these are common to any activity associated with machinery, to which mankind, at least in the so-called developed or industrialized countries, has become ontologically, if not phylogenetically, adapted. Some hazards, such as exposure to forces resulting from rapid acceleration and deceleration, and cosmic radiation at high altitudes [1], are physical or abiotic. Others are physiological, such as increasing hypoxia with increasing altitude [2], which in modern jet aviation is countered by pressurizing the cabin to an altitude of two to three thousand metres above sea level (it would be prohibitively costly to pressurize cabins to sea level). A corollary of this pressurization is the need for cabins to be sealed and supplied with air pumped in from outside. Confining large numbers of unscreened people, some of whom may be infected with respiratory and other diseases, in an enclosed space exposes them to the risk of cross-infection. This biotic hazard has been the subject of a great deal of attention [3,4,5,6,7]. It is partly alleviated by design of the airflows within the cabin—air typically enters from above each passenger seat and is sucked out through vents in the sidewalls just above the floor [8,9]. Since it is rather—albeit not prohibitively—costly to use solely fresh air, typically 50% recirculation takes place and HEPA filters, which retain particles less than about 300 nm in diameter, are installed in the recirculation ducts in order to remove most microörganisms, which mainly originate from the human occupants of the cabin.

## 2. Xenobiotic Hazards

Relatively less attention has been paid to xenobiotic hazards. Typically, these are likely to be molecules much smaller than the particles captured by a HEPA filter (ultrafine particles as a hazard are discussed in Section 6); hence, they are not easy to filter. Molecular filters, with pores only a few nm in diameter, have been reported [10], but systems for molecularly purifying air are typically not filters at all, in the sense of physically retaining molecules by pores smaller than them, relying instead on adsorption to a large surface area. Carbon is a popular adsorbent [11,12]. Such filters may be unable to withstand the high temperature of air emerging from bleed ports in the engine (see Section 3), hence must be incorporated in the recirculation system—typically as a so-called split-bed filter, with the conventional HEPA filter next to the carbon adsorbent [13]. The disadvantage of this arrangement is that the freshly bled-off air is untreated. Ref. [13] compares such filters with several other kinds.

There was early awareness of some molecular hazards, notably the gases carbon monoxide (CO), carbon dioxide (CO_2_) and ozone O_3_) (ref. [14], ch. 5). CO was already a known hazard in general aviation, typically leaking into the cockpit from engine exhaust. Indeed, it is a hazard whenever there is combustion of carbonaceous materials; its formation is expected wherever carbon-containing substances are heated in the presence of oxygen [15]. Although long-considered to be primarily an asphyxiant, the neurotoxicity of CO is now recognized [16]; and many of its symptoms are similar to those of tricresyl phosphate (TCP) poisoning [17]. The asphyxiant action arises through the binding of CO to haemoglobin, and CO can similarly inactivate other haem proteins; since cytochrome P450 (a haem protein) is the primary enzyme involved in detoxifying inhaled or ingested TCP [18,19], there is adverse synergy when both TCP and CO are present [20]. The concentration of CO is mandated to not exceed 50 ppmV (paragraph (b) (1) of ref. [21]).

Since carbon monoxide is endogenously produced in the human body [22], and plays some important physiological rôles, it is not unambiguously a xenobiotic in the sense that the organophosphates tributyl phosphate (TBP, see Section 8) and tricresyl phosphate (TCP, see Section 4) are. For this reason, we shall not devote much attention to it in this paper, but we should be mindful that its presence may constitute at least as great a neurotoxic hazard in the aircraft cabin as does the presence of TCP.

Ozone is likewise endogenously produced [23], hence not strictly a xenobiotic. Its concentration is mandated to not exceed 0.1 ppmV above flight level 270 (paragraph (a) (2) of ref. [24]). At this altitude, the external atmosphere is likely to contain several ppm. Peak concentration, in the range 5–15 ppm, occurs around flight level 1000.

CO^2^ is a major endogenous product [25]. Its concentration is mandated to not exceed 0.5%V (paragraph (b) (2) of ref. [21]).

Ref. [14], ch. 3 covers the development of these regulatory standards.

## 3. Bleed Air and Its Consequences

Jet engines were first developed for military use, and it was a fairly obvious development to bleed off some of the air that was anyway being compressed and concomitantly heated in the engines before the combustion chamber for the purposes of pressurizing the cabin rather than having a separate, inevitably heavy, pump for compressing and heating the air directly from the external atmosphere. Furthermore, air was already being bled off for other purposes such as anti-icing and to provide pneumatic power elsewhere in the aircraft (see Section 5 of ref. [26] for details of aircraft bleed air systems).

The possibility of xenobiotic contamination of bleed air was considered at the time of its introduction [27], albeit with the conclusion that it was not toxic to human beings. It is reasonable to assume that the internal spaces of a jet engine are sterile, hence bleed air avoids exogenous biotic contamination. (Note that jet fuel can host microbes [28], harmless to human beings.) The main source of the xenobiotic contamination was reckoned to be leakage of engine lubricating oil into the air being compressed. The oil, in its lubricating circuit and sump, is confined by oil seals, of which several different designs exist [29,30,31,32], none of which can provide perfectly hermetic sealing, hence some leakage is, essentially, inevitable [26]. Jet engine oil consumption is typically in the range 0.5–2 dm^3^ per hour in flight [26], some of which will be lost through the seals. The corollary of oil loss is the need to top up the oil. Since the oil slowly acidifies [33]—modern jet engine oil is synthesized from polyalcohols and long-chain fatty acids (e.g., [34,35,36]), and slowly hydrolyses—frequent topping up is advantageous for eliminating the acids [33].

In 1977, it was reported that “A previously healthy member of a [military] aircraft flight crew was acutely incapacitated during flight with neurologic impairment and gastrointestinal distress” [37]. The investigation concluded that “The etiology of his symptoms was related to an inhalation exposure to aerosolized or vaporized synthetic lubricating oil arising from a jet engine of his aircraft” [37]. In order to obviate risk from breathing xenobiotically contaminated bleed air, in a small military aircraft, every member of the crew can wear a so-called ‘oxygen mask’ providing clean air from small, portable cylinders.

Large passenger aircraft (jetliners) for commercial civil aviation, such as the B707 (just over 1000 were produced from 1956 to 1978, with some still in service), the DC-8 (just over 550 were produced from 1958 to 1972; since 2013, none appear to be in service), and the VC10 (just over 50 were produced from 1962 to 1970; since 2013, none appear to be in service), pressurized the cabin using air taken directly from the exterior and compressed and heated with dedicated machinery. The engineering advantages of bleed air were subsequently recognized and adopted in civil aviation and from the 1970s onwards, all large passenger jet aircraft used bleed air, until the introduction of the B787 (it entered commercial service in 2011 and more than 1250 have been manufactured to date), which (alongside many innovative design features) reverted to dedicated compressors for the cockpit and the main passenger cabin, but so far no other designs have followed suit. Surprisingly, the gradually increasing bypass ratio of the turbine, which greatly increases the efficiency of the engine, was not accompanied by any reconsideration of the economics of bleed air, which is actually rather costly for high-bypass-ratio engines, and it would be advantageous to bleed the air off the bypass [38], with the collateral advantage of significantly less contamination. So far, this remains only an engineering proposal, however. Table 1 compares bleed-air with bleed-free architectures.

So much for some of the engineering aspects. In the long history of regulation of cabin air contamination (discussed in Section 2), only ozone, carbon monoxide and carbon dioxide have been explicitly named. But paragraph (b) of ref. [21] mandates that ‘crew and passenger compartment air must be free from harmful or hazardous concentrations of gases or vapors’. Of course, this statement begs the question, what gases or vapors are harmful or hazardous, and at what concentrations? This paper focuses on the industrial chemicals associated with bleed air in order to answer it.

## 4. Fume Events and Pilot Incapacitation

Since around 1990, there have been some alarming incidents of sudden pilot incapacitation at critical phases in flight (such as during the approach to landing). A thoroughly investigated incident was that at Malmö in 1999 [39]. The conclusion was “The incident was caused by the pilots becoming temporarily affected by probably polluted cabin air”. Modern jet engine lubricating oil is mainly (>95%) composed of synthetic esters of polyalcohols like pentaerythritol and long-chain fatty acids [40,41] (specific formulations are closely guarded commercial secrets of the manufacturers). A crucial ingredient (comprising ∼3%) is tricresyl phosphate (TCP), required for its antiwear function [42]. TCP is very well known as a neurotoxin, above all thanks to the ‘Ginger Jake’ scandal during the prohibition era in the USA, which resulted in tens of thousands of people who had inadvertently ingested TCP becoming partially crippled. There have been numerous other cases of accidental poisoning since then, mostly oral [43,44,45]. In general, oral toxicity is less severe than inhalation toxicity, which has caused toxic injury to workers in factories producing TCP [46,47].

Also in the 1990s, the phenomenon of ‘fume event’—when the aircraft cabin is filled with smoke (although there was at the time no regulatory definition)—started to be noticed. Typically, it occurs when there is a catastrophic failure of an oil seal [12]. In 2006–7, Murawski and Supplee collected and critically examined Service Difficulty Reports (SDRs) and Accident and Incident Data System (AIDS) reports filed with the US Federal Aviation Administration (FAA). They collected 350 reports pertaining to fume events over an interval of 18 months; in addition, they collected 115 reports from flight attendants to their airlines, and another 37 reported in newspapers, with only partial overlap between the categories, hence evidencing some underreporting to the FAA [48]. The reports in the final list pertained to contamination of the aircraft cabin air supply; i.e., distinctly different from other smoke-producing events, such as galley fires, conflagration of interior furnishings, and heating or ignition resulting from an electrical fault, which generate smokes of a composition not unique to the aircraft cabin interior. An update appeared in 2014 [49]. In 2011, an FAA ‘Information for Operators’ circular mentions 900 fume events annually [50], apparently indicating a tendency to increase compared with the Murawski and Supplee report from five years earlier. In the same year, Murawski presented an analysis of fume events over two years at a major US airline [51]. Lind has also gathered some examples of fume events (Appendix to ref. [52]). Appendix D of the thesis by Hildre and Jensen lists 625 events globally for the seven years 2007–13 [53]. According to recent reporting by the *Wall Street Journal*, the rate of occurrence of fume events in 2024 was about an order of magnitude greater than a decade earlier [54,55]. In response to the passing of the FAA Reauthorization Act of 2024, which itself evinces legislative concern about the matter, the FAA issued new guidance on the voluntary reporting of fume or smoke events [56].

**Figure 1:**
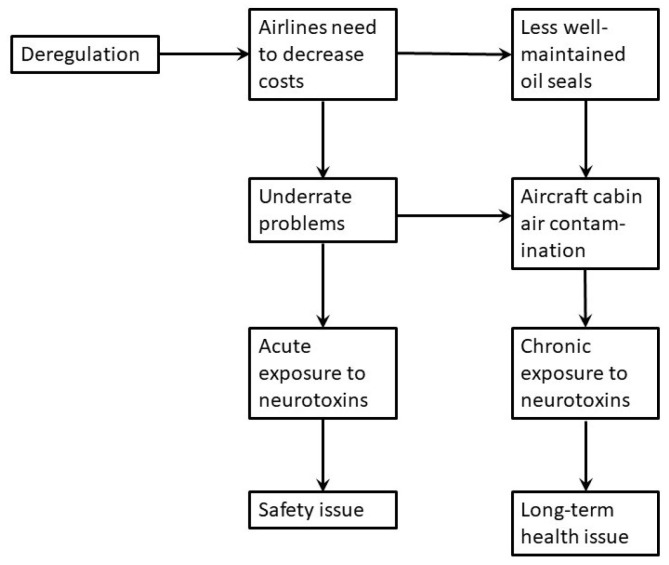
Diagram of immediate effects (causal diagram) relating to fume events and their consequences.

Two principal reasons for the growth of oil seal-related fume events—i.e., those arising from contamination of the bleed air supply—may be suggested. Firstly, there is the exponential growth of civil aviation by 4–5% per annum since its inception. This is, however, evidently insufficient to alone account for the increased frequency of fume events. Secondly, there is the strong commercial pressure since deregulation of the airline industry in 1978 to keep aircraft maintenance costs as low as possible consistent with safety with respect to reliable delivery of engine thrust [52,57,58,59]. Before deregulation, engines were overhauled after a fixed number (typically 5000) of flight hours. It was subsequently found that, thanks to continually improving technology, engines could continue working without overhaul for up to ten times longer, and the concept of engine health management (EHM) became standard practice [41]. Apparently, oil seals degrade faster than thrust-critical components; oil seal integrity is not essential to thrust delivery, and it is too expensive to change an oil seal other than during general engine overhaul for the seals to be changed with the frequency needed to ensure the absence of fume events (of course, they ought to be replaced, at considerable expense, following a fume event). Furthermore, as pointed put in Section 3, the frequent topping up required to compensate for oil loss helps to keep the oil in good condition. Figure 1 captures the supposed causal relations leading to health and safety issues.

## 5. The Principal Hazards

The concentration of TCP in aircraft cabin following a fume event is estimated [60] to well exceed the legally binding workplace exposure limit (WEL) published by the UK Health and Safety Executive (HSE) [61], but an aircraft cockpit or cabin appears to be outside the scope of the WELs (¶62 states that they ‘relate to personal exposure to substances hazardous to health in the air of the workplace. The limits cannot be adapted readily to evaluate or control non-occupational exposure…WELs are approved only for use where the atmospheric pressure is between 900 and 1100 millibars’—an altitude of 7000 feet (to which aircraft cabins are typically pressurized) has an air pressure of only about 700 hPa or mbar)—the cockpit and the cabin are, of course, the regular workplaces for aircrew, and appropriated as such for those passengers who work during a flight, but many passengers do not. No explanation of the pressure limitation is offered in the EH40 document. The relationship between concentrations expressed as a mass per unit volume and ‘ppm’ (or ‘ppb’) is, of course, pressure-dependent, as is human ventilation [62]. A complicating feature is that CO_2_ concentration tends to be somewhat elevated in the aircraft cabin as the air is gradually enriched in CO_2_ due to human exhalation and is partially recirculated; this elevation affects human ventilation [63].

Formally, any substance listed in a compilation of WELs (such as [61]) constitutes a hazard. Nevertheless, the very concept of a threshold concentration implies that exposure to a concentration less than the threshold is not a hazard. Risk is then the product of some quantitative measure of the adverse effect with the probability of exposure above that limit (or the convolution of the adverse effect with the probability of exposure, both as a function of ambient concentration). For T*o*CP, the World Health Organization has affirmed that ‘Because of considerable variation among individuals in sensitivity to TOCP, it is not possible to establish a safe level of exposure’ ([64], Section 10.1), invalidating the concept of a threshold for that substance. One might reasonably ask where it comes from. Although the earliest limits were established by experimental observations of effects on human beings [65], ethical considerations would generally preclude such experiments; hence, subsequently, they were established via epidemiological observations and experiments with animals, and the actual levels are sometimes rather arbitrary [65]. Evidence for the hazardous nature of TCP is rather extensive (Section 4), but the present WEL (for T*o*CP) needs to be taken *cum grano salis*. As has recently been noted [66], if any adverse consequences of poisoning are irreversible or irreparable, then there can be no lower limit of safety.

When the main engines are not running, the cabin is pressurized from the auxiliary power unit (APU), typically located at the rear of the aircraft. (At some air terminals, a ground support unit is available to supply electrical power and air [from conventional compressors] to the aircraft.) Air is bled off the APU in the same way as off the main engines, hence is subject to qualitatively the same contamination, but quantitatively, it is likely to be worse because, the APU not being as safety-critical as the main engines, it is not generally maintained to the same standard. Furthermore, since hydraulic fluid (the major constituent of which is tributyl phosphate [67]) leaking from the servo systems [68] tends to move to the rear of the aircraft, being picked up by the APU is the most likely route of its ingress into the cabin. The bleed air-free B787 has a special APU (Pratt & Whitney APS5000) that does not supply bleed air.

## 6. Hazards from Abradables and Oil Decomposition

A further xenobiotic hazard in bleed air is the presence of ultrafine particles [69]. While the lubricating oil, considering the high boiling points (over 300 °C [70]) of its constituents, is likely to condense into an aerosol by the time it reaches the aircraft cabin [71], rather than remain a vapor, there is an additional source of particles, namely, the abradable materials with which the extremities of the turbine blades in the engine are coated to improve the fit of the blades to the casing without risk of incursion [72,73]. A typical material is boron nitride in an aluminum–silicon matrix [74]. Aluminum and silicon particles, and particles of several heavy metals, have been detected in aircraft exhaust [75]. In essence, these ‘abradables’ are advanced materials applied to the extremities of turbine blades to perfect their aerodynamic sealing with the wall of the compression chamber, and are deliberately designed to be abradable in order to cope with very slight profile variations without damaging the fixed structures. As with many types of nanoparticles [76,77], in view of their expected composition, the particles derived from abradables would be toxic if inhaled. A recent French court case refers to laboratory findings of metallic nanoparticles in the tissues of a pilot comparable to those found in the cockpit [78].

Since circulating lubricating oil becomes very hot—at least hundreds of °C [79]—attention has been paid not only to its possible volatilization but also to its decomposition, either by burning or pyrolysis (TCP itself is considered to be non-flammable). Decomposition of at least some of the constituents may indeed occur before vaporization [70]. Early, relatively unsophisticated, experiments with heated oil clearly demonstrated the toxicity to laboratory animals of the fumes given off [80,81], without any attempt to identify individual compounds. Rather surprisingly, for many decades thereafter, there seems to have been little attempt to extend this work [82,83,84]. Possibly ethical considerations acted as a brake. Only very recently has the subject been taken up again in vitro, using cultured lung cells [85], and primary cortical rat cultures [86]. Fumes generated from hydraulic fluid, which is mainly constituted from tributyl phosphate, were found to be more toxic to lung cells than tricresyl phosphate fumes. Fumes from TBP, and from triphenylphosphate (TPhP), were found to diminish neuronal activity with a potency comparable to that of TCP, even though TCP is a well-known neurotoxin that engenders demyelination [87] whereas TBP is considered to be relatively nontoxic; cf. the workplace exposure limit is 50 times higher for TBP than for T*o*CP [61]. This may reflect the provisional nature of the WELs as much as anything else. While the mechanism of T*o*CP neurotoxicity is well established (see Section 9), TBP is known to be neurotoxic to zebrafish larvae [88], *inter alia* via neurotransmitter system disruption. Here, it is pertinent to recall that trimethylolpropane phosphate (TMPP) potently inhibits GABA receptors [89], and can be formed from TPhP in situ in jet engine oil [90].

## 7. Aerotoxic Syndrome

Pilot incapacitation incidents and an accumulation of reports [91] of passenger malaise prompted the proposal of ‘aerotoxic syndrome’ [92,93] as a definable medical phenomenon deserving the attention of civil aviation authorities. In 2004, the Safety Regulation Group of the UK Civil Aviation Authority (CAA) undertook research into the toxicity of lubricant pyrolysis products and their deposits in aircraft air conditioning ducts [94]. That there was at that time still a very poor understanding of aircraft cabin air toxicity due to xenobiotics is well illustrated by the final page of that report, in which the lowest dose of TCP resulting in organophosphate-induced delayed neuropathy (OPIDN), which is one of various physiological responses to TCP that have been observed, experimentally established in chickens, is extrapolated to the weight of a human being, with the conclusion: ‘An average man would therefore be able to ingest 7 metric tonnes of pyrolysed oil per day for 74 days without effect’. This conclusion is left without further comment. In any case it has subsequently been realized that the aetiology of aerotoxic syndrome is not congruent with OPIDN [95].

In 2005, the British Airline Pilots Association (BALPA) organized the ‘Contaminated Air Protection’ conference in London, at which a considerable range of apparently occupational pathologies among pilots and cabin crew was evidenced [96]. The Australian Parliament held an enquiry in 1999–2000 into aircraft cabin air contamination, the UK Parliament did so in 2002 and 2007, and in the USA, the FAA established the National Air Transportation Center of Excellence for Research in the Intermodal Transport Environment (RITE), encompassing the Airline Cabin Environmental Research (ACER) programme, which has produced a number of reports on the problem (e.g., refs. [97,98,99]).

## 8. Measurements of Cabin Air Contamination

The results of the UK parliamentary enquiries prompted the commissioning of more extensive studies by the UK Government: the ‘Aircraft Cabin Air Sampling Study’ (ACASS) undertaken by Cranfield University, and the ‘Cabin Air–Surface residue study’ (CA-SRS) undertaken by the Institute of Occupational Medicine (Edinburgh). The ACASS report, published in 2011 [100], found TCP and particulates (the nature of which was not characterized) during flight, as well as other contaminants including toluene, tributyl phosphate (TBP), limonene and carbon monoxide. Toluene, a rather widely used industrial solvent, was presumed to originate from engine cleaning. As a neurotoxin, its presence in aircraft cabins deserves attention. TBP is the major component of hydraulic fluid, and its ubiquity suggested that leakage from the hydraulic control system is endemic. Although it is an organophosphate differing from TCP only inasmuch as the aryl substituents of the phosphorus are replaced by alkyl substituents, its toxicity is considered to be rather low (but cf. the in vitro studies mentioned in Section 6). Limonene is widely used to perfume domestic cleaning formulations.

The ACASS study, and measurements carried out specifically in the cockpit [101], did not reveal any exceedance of the mandated limit for carbon monoxide.

The results of the ACASS study were extensively discussed at a conference convened at Cranfield University later in 2011 [102]. Two important deficiencies were established: (1) although the objective of the study was to measure actual contamination levels during a fume event, no fume event actually occurred during the 100 test flights of the study; (2) the measured concentrations of TCP and the other contaminants were compared with WELs although, as mentioned above (Section 5), the aircraft cabin does not fall in the scope of the WELs. Nevertheless, the fact that the measured levels of contamination were lower than the WELs was interpreted as indicating that the levels were safe: indeed on the day the report was published (10 May 2011) by the Department for Transport, the Minister of State (Mrs T. Villiers) gave a written statement in Parliament that ‘The main conclusion of Cranfield’s research was that there was no evidence of pollutants occurring in cabin air at levels exceeding available health and safety standards’ [103] (later echoed by Attlee in the House of Lords [104]). Crump et al. [100] did not actually state that this was the main conclusion (although its position at the very end of the main body of their report might be taken to imply it), and they referred to ‘guidelines’, whereas Mrs Villiers referred to ‘standards’, which are not quite the same thing. The ACASS study did not refer to the World Health Organization (WHO) statement, ‘because of considerable variation among individuals in sensitivity to T*o*CP [tri-*ortho*-cresyl phosphate], it is not possible to establish a safe level of exposure’ [64]. Besides, at the time, the cabin surface residue report had not been published; it finally appeared in 2012, reporting that extensive deposits containing TCP were found throughout aircraft cabins and, especially, in the ducting carrying the bleed air from the engines into the cabin [105]. These results have been corroborated by extensive observations reported by Scholz [106].

Regarding the actual measurements [100], these have also been subjected to various technical criticisms [107], one conclusion of which is that the concentrations of the xenobiotics are likely to have been underestimated. There have been other studies of aircraft cabin air contamination, already reviewed by the present author [108]; these and a later one are summarized in Table 2. This last one [109] had the merit of including flights with the B787, which lacks the bleed-air architecture. Refs. [110,111] are sometimes cited but did not include TCP concentrations as measurands.

Undoubtedly, such studies are not easy to carry out. The favored approach is to collect the air in pumped tube samplers, from the adsorbents of which the contaminants are subsequently desorbed and analyzed using gas chromatography and mass spectrometry. An overarching weakness of all the studies is the failure to record engine details, including maintenance records. This presents an immediate obstacle to understanding the underlying reasons for the considerable variation between the results.

A further weakness of most of these studies is the failure to investigate the relationship between air contamination and symptom reporting, as has been pointed out by Harrison and Mackenzie Ross [113]. A notable exception is van Netten’s study of BAe146 aircraft [12], but it did not look for TCP.

Given that small portable air samplers have been invented and described in detail [114,115,116], it is surprising that they have not been widely deployed to gather data. The database is still very small, especially with regard to the continuing exponential growth of aviation, and the significant increase in the number of fume events [54,55], for which we still have no measurements but must rely upon an estimate [60]. Chemical sensors have been discussed in refs. [97,117]; they would have the advantage of yielding continuous measurement results in real time, but do not yet appear to be reliable enough for deployment in measurement campaigns.

## 9. Mechanisms of TCP Intoxication and Detoxification

A major, questionnaire-based, survey of a large number of Dutch frequent flyers pointed out that the results were consistent with either of the two extreme scenarios: (1) the cabin air in 50% of flights is contaminated with neurotoxins, to which everyone is susceptible; or (2) all flights are contaminated, and up to 50% of the population is susceptible to contract adverse effects from inhaling the contamination [118]. The data from [100] indicated that only 25% of flights were contaminated with TCP, which, if representative of passenger airlines, rules out both scenarios. Regarding susceptibility, sequencing of the human genome [119] ushered in a new era of genomic medicine and the question naturally arises whether there is a genetic component of susceptibility to aerotoxic syndrome, since the majority of the population appears to be unaffected [66]. Progress in establishing the mechanisms—there appear to be several possible ones—of the action of TCP in the body has been slow. There is peripheral neuropathy (axonopathy), but as Tkachuk and Matiytsiv have recently pointed out [45], its precise mechanisms are still poorly understood. It has been emphasized by Howard et al. that the aetiology of aerotoxic syndrome is far from clear and not congruent with OPIDN [95], which is well established as the endpoint of oral toxicity [120] (cf. Section 4). If the neurofilaments in axons are degraded, which seems to be one of the results of administering TCP, intracellular transport is disturbed. Axonal degeneration has been observed in one of the rare autopsies of an airline pilot [121], and such degeneration can be expected to have wide-ranging and far-reaching cognitive consequences, as have been observed in airline pilots [122]. These findings are corroborated by noninvasive functional magnetic resonance imaging of the brain in parallel with cognitive assessment of aircrew [123].

Two important milestones were: (1) the elucidation of the cytochrome P450 enzymes responsible for the primary detoxification [124], namely, 1A2 and 3A4, which convert TCP to 2-(o-cresyl)-4H-1,3,2-benzodioxaphosphoran-2-one (CBDP; also known as cresyl saligenin phosphate) [19]; and (2) the discovery that butyrylcholinesterase (BChE) circulating in the blood serves to scavenge all isomers of TCP [125,126,127,128]. This plays an important rôle in TCP toxicity, because in careful studies using pure isomers, Henschler established that only the isomers containing at least one *ortho* substituent are neurotoxic [129]. The subsequent work of Casida made it clear that an *ortho* substituent is required for cyclization of oxidized TCP to form CBDP. If the affinity of BChE is only slightly higher for non-*ortho*-isomers than for the *ortho*-containing isomers, BChE will become saturated upon exposure to the usual mixture in which the *ortho*-free isomers are in great excess, allowing uncomplexed *ortho*-isomers to reach and be metabolized in the liver. Hence, counterintuitively, a mixture of *ortho-* with non-*ortho*-isomers may actually be more neurotoxic than pure *ortho*-cresyl phosphate, which would be effectively scavenged by BChE if they did not have to compete with the non-*ortho*-isomers. Hence, the strenuous efforts to diminish the *ortho* content made after the realization that neurotoxicity was solely associated with the *ortho*-isomers may not have directly alleviated the problem. This is quite apart from the observation that the proportion of *ortho-* to non-*ortho*-isomers measured in the cabin air is much greater than the proportion in the oil [130], an observation for which transisomerization has been eliminated as a cause [131].

It was long accepted that CBDP was a potent neurotoxic substance, but quite recently Hausherr et al. have shown that both CBDP and *ortho*-containing TCP have distinct neurotoxicological actions [132]. On the other hand, the lack of neurotoxicity of the *meta-* and *para*-isomers found by Henschler has been confirmed by more recent work [45]. CBDP is reactive, hence quite labile, and may function as a general phosphorylating agent; given the importance of phosphorylation in cell metabolic regulation, this could well account for the variety of effects of TCP intoxication [45]. Experiments with rodents have not, however, established any carcinogenic action [133].

Naringenin [134] is a potent inhibitor of cytochrome P450 [135], and its relevance to TCP intoxication has already been pointed out [135]. A specific dietary source of naringenin is grapefruit. Therefore, knowledge of a subject’s grapefruit consumption would appear to be indispensible for understanding response to TCP.

## 10. Risk of TCP Intoxication

Clearly, TCP constitutes a neurological hazard. The question is whether typical exposures in aircraft are such as to convert the hazard to a risk. To answer it, let us look at the ostensive evidence for neurological injury.

Narrative evidence from affected subjects (aircrew) is widely found (e.g., [52,136]). In some cases, medical examinations have been undertaken [37,122,123,137]; see also several papers from the BALPA conference already mentioned [96]. The results from an autopsy have already been cited [121]. Nor is the problem confined to aircrew. Frequent flyers have also narrated their problems (e.g., [138]). Even a single flight can cause a problem [55]. One of the present difficulties is establishing whether, on any given flight, exposure to TCP and other neurotoxins actually took place. Such measurements as have been carried out (Section 8) revealed detectable concentrations of TCP on about 25% [100], 15% [112], and 31–64% for non-B787 flights and 55–84% for B787 flights [109]. This last result is surprising because the B787 does not pressurize the cabin with bleed air. The large and overlapping ranges of occurrence for the two categories of aircraft suggest that the difference is not significant; hence, without careful scrutiny of the entire report [109], little weight should be attached to this result. The authors themselves state that further research is needed to determine the origin of the TCP, possibly using ^13^C-labeled TCP.

A much larger research project, the ‘Fresh Aircraft–Contaminated Cabin Air Toxicity Study’ (FACTS), began in 2016 and was closed in 2019. Its primary aim was to characterize the composition and concentration of pollutants in an aircraft cabin following engine oil leakage using a test bench simulating the bleed air system (the Bleed Air Contamination Simulator, BACS). Toxicology experiments, focusing on inhalation neurotoxicity and behavioral changes, used zebrafish and mice, but in the end, disappointingly few experiments could actually be undertaken. Two flight tests were carried out but the reports were, and remain, designated confidential. The final report (which includes summaries of the confidential reports) is an extensive document (the summary section alone occupies 15 pages) [139], which will doubtless repay careful study, beyond the scope of the present paper.

Regrettably, and as already pointed out, all such reported surveys have so far failed to record essential technical information about the aircraft and, especially, the engines, such as time since last overhaul. Hence, at present, it is not possible to correlate the occurrence of TCP with specific technical characteristics, although certain aircraft were unambiguously found to be more prone to contaminate the cabin air—for example, according to [100], the BAe 146 (about 500 built, of which about a hundred are still flying) and the B757 (about 1000 built, of which about 200 are still flying) had the most contaminated cabin air among those aircraft tested. Furthermore, analysis of environmental conditioning duct residues unambiguously reveals the accumulation of TCP in aircraft with bleed air [94,105,106]. Therefore, we can reasonably assert that the presence of TCP in aircraft cabin air has been established in a statistical sense.

A blood test revealing recent TCP exposure was carried out on 12 airline passengers (on different flights) [140]. Evidence for very low exposure was found in 6 of them. None of the 12 had reported any adverse health symptoms. As well as variation of exposure, we are also confronted with the difficulty of variation of individual susceptibility. There is enormous evidence for biochemical individuality [141], not least regarding the liver and its xenobiotic detoxification capacity. The variation is partly due to genetic differences, but is also ontological, especially regarding the important cytochrome P450 detoxification apparatus [142], which comprises a vast family with many different members, the characteristics of which partly depend on the history of past xenobiotic exposures. The WHO International Programme on Chemical Safety (IPCS) considers that ‘because of considerable variation among individuals in sensitivity to T*o*CP [tri-*ortho*-cresyl phosphate], it is not possible to establish a safe level of exposure’ [64].

The TCP exposure of the deceased pilot subjected to an extensive autopsy [121] was estimated from his flight records, assuming that airline flights constituted the sole source of exposure (there was no evidence for other significant exposure) [143], with the conclusion that ‘the estimated exposure to tricresyl phosphates of the neurologically injured pilot is considerably smaller than current paradigms would suggest is sufficient to have caused his neural degeneration and associated problems’. ‘Current paradigms’ presumably means the WELs so strongly emphasized in ref. [100], but the IPCS already noted that it was not possible to establish a safe level of exposure [64]. Daughtrey et al., in a study with hens, found that subchronic TCP exposures did not result in neuropathology, albeit that the study was focused on OPIDN [144].

Other possible factors that could lead to the symptoms of aerotoxic syndrome need to be carefully considered. CO has already been mentioned. Another is diet. This has already been discussed in connexion with ‘airsickness’ [145] (note that airsickness is not the same as aerotoxic syndrome, although it might overlap or encompass it). One interesting finding was that a diet of food high in thiamine (vitamin B_1_) appeared to promote airsickness. On the other hand, thiamine deficiency is recognized as a cause of Wernicke’s encephalopathy, the characteristic lesions of which may resemble those of organophosphate poisoning [146], and its symptoms overlap with those of aerotoxic syndrome [147,148,149].

## 11. Alleviating the Risks

The passing of the US FAA Reauthorization Act of 2024 (in May 2024), championed by Senator Richard Blumenthal, which mandates the ‘Installation onboard aircraft of detectors and other air quality monitoring equipment (Section 362 CABIN AIR SAFETY (d) (3); a fume or smoke events is defined (e) as ‘an event in which there is an atypical noticeable or persistent presence of fumes or air contaminants in the cabin, including, at a minimum, a smoke event’) seems to indicate an affirmative response to the question ‘Is it time to act?’ [150]. Assuming that the presence of different substances and their concentrations can be distinguished by the air quality monitoring equipment, it should greatly assist the establishment of a causal relation between exposure and aircrew and passenger ill health (that the Act considers to be a safety issue). Without identification of contaminants, it would be difficult to provide biomedical corroboration of the onset of ill health. The possibility of such a causal relation has long been a preoccupation [151], without much progress in establishing it; an analysis of extant data revealed no more than the incorrectness of what was called the ‘Attlee hypothesis’—‘We do not have evidence that passenger health is affected by fume events’—without it being possible to assert the contrary [151]. Once air quality monitoring equipment is routinely used, presumably a body of data sufficiently large to indicate an association between air contamination, if it exists, and ill health will be built up. Such an association, if found, would encourage the collection of pre- and post-flight records of symptoms and measurements of physiological indicators from individual flyers.

Some will argue that the evidence of contamination-induced ill health is already sufficient to warrant application of the precautionary principle [152]. If so, how should it be applied? Complete cessation of aviation might seem too extreme a response (although such cessation, by the year 2050, is deemed necessary in those jurisdictions that have enacted mandatory ‘net zero’ legislation [153]). A milder response would simply be to emphasize *caveat volator*, much as the highway traveler should be mindful of *caveat viator* as an enjoinder to exercise due care in order to detect and avoid hazards [154].

## 12. Assessing Reasonable Mitigating Actions

As the preceding section has shown, it looks like a difficult problem to rationally decide what measures, if any, it is worth taking to improve aircraft cabin air. On the one hand, it would obviously be desirable for the air to be as pristine as that available in a rural terrestrial location; on the other hand, given the technical nature of aircraft cabin ventilation, it is costly to improve air quality, which is presumably why the contamination problem largely persists.

A useful approach for balancing these two aspects is based on the life quality index *Q*, which is, roughly speaking, the product of annual income *G* with life expectancy *X*. The most it is worth spending on a health-improving measure is that amount for which the decrease in income required to pay for the measure just balances the resulting increase in longevity in terms of *Q*. In our case, the health-improving measure is the elimination of bleed-air contamination, such as by retrofitting air purifiers, or adopting bleed-free cabin pressurizing designs for all new aircraft. To make a quantitative assessment, we need to know the effect of cabin air contamination on longevity. This idea has been applied to assessing the cost–benefit ratio of measures to improve environmental air quality [155] and a reasonable price for a medicinal drug [156,157]. In early applications [158], future income was discounted, and life expectancy was raised to the power of the work–life balance; later the discounting was dropped and life expectancy was raised to the power of 1−ε, where ε is risk aversion (or, equivalently, the negative of the elasticity of marginal utility of income) [159,160]. In developed countries, ε has been estimated as 0.91 [161].

Let us define

(1) $$Q=G^{1-\varepsilon }X\;,$$

and then apply a small perturbation (e.g., some small expenditure −δG causes a small increase in *X*), obtaining

(2) δQ=−(∂Q/∂G)δG+(∂Q/∂X)δX,

from (1), we get

(3) $$\delta Q=-\left(1-\varepsilon \right)G^{-\varepsilon }X\delta G+G^{1-\varepsilon }\delta X\;.$$

We divide this expression by (1) to obtain

(4) δQ/Q=−(1−ε)δG/G+δX/X

and as the left-hand side tends to zero (Q≠0), after rearrangement, we obtain an expression for the sensible spend, namely,

(5) $$\delta G=\frac{G\delta X}{(1-\varepsilon )X}\;.$$

The J-value is defined as the ratio of the actual spend $\delta \hat{G}$ to the sensible spend:

(6) $$J=\delta \hat{G}/\delta G$$

or, writing it in full with sensible spend *per capita* $\delta \hat{G}/N$, where *N* is the relevant population size:

(7) $$J=\frac{\delta \hat{G}\left(1-\varepsilon \right)X}{NG\delta X}\;.$$

It often seems to be tacitly assumed that the reason nothing is done to improve aircraft cabin air is that it would cost ‘too much’, but even a rudimentary attempt to assess the cost does not appear to have been published yet. Let us apply Equation (7) to investigate the economics of mitigation, and let us assume that a single, initial spend $\delta \hat{g}$ on the mitigating measure is dominant. In this case, we have [156]:

(8) $$\delta \hat{g}=\delta \hat{G}/X\;.$$

In order to see how much expenditure is justified for J=1 (i.e., the amount it would be sensible to spend in order to maintain the life quality index at its present value), we rewrite Equation (7) using (8) to obtain

(9) $$\delta \hat{g}=\frac{NG\delta X}{1-\varepsilon }\;.$$

The UK has a total population of about 68 million. Let us suppose that 1% of the population can be considered as frequent flyers (including aircrew), hence N=680,000. For *G*, we take gross domestic product (GDP) *per capita*, presently about GBP 40 k. There is no statistical evidence of increased mortality among aircrew [162], hence we would not expect it among frequently flying passengers; let us therefore use morbidity rather than mortality. On this, there is very little statistical information, but undoubtedly among at least some passengers, frequent flying leads to an increased morbidity [138], and similarly with aircrew [136], and in both these cases, the subject was unable to continue working.

Inserting *N*, *G* and ε into Equation (9), we obtain 302 GGBP per year of extension of life free from debilitating morbidity. If replacing bleed air-pressurized aircraft with bleed-free ones would mitigate the hazard to the extent that aircrew and frequent flyers would enjoy one extra year of good health on average, then it would be worth purchasing approximately 1200 B787s, which cost about 250 MUSD each; this number is roughly equivalent to the number of aircraft currently operated by UK-based airlines.

This straightforward calculation clearly shows that mitigation is affordable; essentially, anything that falls below the J=1 threshold pays for itself through improved health outcomes. Moreover, cheaper solutions may emerge upon further investigation, such as the proposal in ref. [38], or the filters described in [13]. Furthermore, many of the aircraft operated by UK-based airlines are smaller than the B787, hence could be replaced by smaller bleed-free aircraft. The assumptions and quantities in the calculation can of course be discussed and refined, but already this first result shows that there is no fundamental economic barrier to eliminating aircraft cabin air contamination.

There is one significant difference between this case and the various ones considered in ref. [157]: the latter all concerned expenditure by national or nationalized industries such as Network Rail or the National Health Service, whereas the former concerns private companies, to which the life quality gains through improved health of aircrew and passengers do not directly accrue. There is, however, a complex web of interactions between the state and (privately owned) airlines; one need only mention the absence of duty on jet fuel (for commercial flights), and the air passenger duty levied on each ticket, which often amounts to more than the actual fare. Hence, this difference does not present a fundamental obstacle to mitigation.

## 13. Conclusions

Table 3 summarizes the two principal xenobiotic hazards and risks in aircraft cabin air. The WELs are of course only indicative because, as discussed above, they are not applicable to the conditions in an aircraft cabin in flight. The maximum concentration of T*o*CP is well below the WEL under normal flight conditions, but is estimated to exceed it fivefold in a fume event. A similar situation obtains with the ultrafine particles measured in the aircraft cabin.

These numbers suggest that for a presumed majority of the population, normal flights may pose no special risk. For a presumed significant minority, who may not even be aware of their susceptibility, normal flights evidently do present a risk of intoxication, and it would appear to be a sensible precaution to fly in a bleed-free aircraft (such as the B787). If a fume event occurs, there may be a health risk to a majority of the population. The size of the susceptible minority can be estimated from the data in ref. [118] (about 50% of flyers suffer ill-health after a flight) and from the knowledge of the proportion of flights that are contaminated (Section 10). This proportion is unfortunately not known very precisely. If one takes it as 70%, which is around the upper end of the range of estimates, one requires a susceptible fraction of about 70% in order to be consistent with ref. [118]. This is a ‘worst-case’ scenario that assumes all the reported illness is caused by air contamination. If, in reality, only half of it is (and it could indeed be a smaller fraction), and we take the proportion of flights with contaminated air as 0.5, then we require a susceptible fraction of about 50% to be consistent with the survey data. Clearly, there are still significant knowledge gaps in this area. One such gap is whether any independent assessment of susceptibility is possible, e.g., via genetic testing. This would of course be of great practical use for individual flyers. Another gap is the correlation of symptoms with exposures. No correlation emerged from the small dataset assembled by Liyasova et al. [140]. Since most in-flight measurements of cabin air contamination did not record symptoms from aircrew (and passengers if present—in most cases, they were not), there is still very little statistical information associating contamination with ill health.

We have, therefore, the somewhat paradoxical situation of a significant number of flyers reporting indications of ill health during or after a flight, even in the absence of a ‘fume event’, indications often centred on neurological complaints, with symptoms grouped under the ‘aerotoxic syndrome’ descriptor; while in-flight measurements (albeit not during fume events) have revealed the common presence of neurotoxins (most notably TCP and CO) in cabin air, but at concentrations too low to cause injury according to current understanding. To resolve the paradox, several possibilities can be suggested:

- Current understanding is wrong; chronic low exposures can cause injury, at least to susceptible individuals;
- While exposure to individual neurotoxins is indeed below putative thresholds for injury, they are above threshold in combination (a particularly pertinent one is that between TCP and CO [20].

While it would be a straightforward piece of experimental work to subject large numbers of individuals to low doses of, especially, TCP and CO, individually and in combination, and record symptoms, this might be considered unethical (which constitutes another paradox, because the doses would be below official toxicity thresholds). Such an investigation should also encompass genetic examination in order to elucidate the basis of differences in susceptibility, and should take due account of other possibly relevant factors such as vitamin B_1_ deficiency and naringenin consumption, and the mildly hypoxic conditions of the aircraft cabin.

Figure 1 suggests that simply by reintroducing a strict regulatory regimen, the problem of xenobiotic cabin air contamination could be largely resolved without any new technologies. It would be accompanied by a return of airline ticket prices to prederegulation levels. The flying public is already being prepared for a substantial increase in ticket prices due to the introduction of sustainable fuels [166]. On the other hand, continuing progress in advancing prognostics and health management (PHM) may lead to innovative cost-effective solutions to the oil seal maintenance problem [167]. Whatever the measure adopted, the calculation presented in Section 12 indicates that a solution to eliminate ill health among flyers is affordable.

When only a tiny fraction of the population traveled, voluntarily, on airliners, either as passengers or crew, the hazards were not at the forefront of attention. Nowadays, after decades of exponential growth (around 4% per annum), a significant fraction of the population may now be exposed to the hazards of contaminated aircraft cabin air, hence attention to the problem is warranted, and is occurring [168]. It seems appropriate to conclude with a quotation from Sherk: ‘The costs of not doing so [i.e., of not providing clean cabin air in the present context], especially if low levels of exposure over long periods of time result in significant neurological or reproductive injury, may be politically, legally and economically unacceptable’ [169].

## Figures and Tables

**Table 1:** Advantages and disadvantages of bleed air for cabin pressurization.

Advantages	Disadvantages
Engineering convenience	Weakens thrust
(fewer rotating parts)	Greater fuel consumption
	Xenobiotic contamination

**Table 2:** Summary of measurements of tricresyl phosphate (TCP) in the cabin air of commercial airliners. Concentrations are given in μg m^−3^.

Ref.	Maximum	Mean	Median	No Flights
[100]	38	0.22		100
[98]	nd ^*a*^	nd		83
[112]	0.246 ^*b*^		0.090	90
	0.217 ^*c*^		0.041	
[109] ^*d*^	1.515	0. 009	0.002	61
[109] ^*e*^	0.403	0.02	0.005	8

^*a*^ Not detected. ^*b*^ For the start phase. ^*c*^ For the entire flight. ^*d*^ Not including B787 aircraft. ^*e*^ B787 aircraft only.

**Table 3:** Summary of xenobiotic hazards and risks in the cabin air of commercial airliners pressurized with bleed air; workplace exposure limits give an indication of hazard (potential for harm), and the measured quantities give an indication of what is actually encountered. All concentrations are given as μg m^−3^.

Substance	Max. Conc. in Cabin Air [100]	Estimate in Fume Event [60]	8 h WEL [61]
Tri-*ortho*-cresyl phosphate (T*o*CP)	23	500	100
Respirable amorphous silica dust ^*a*^	560	13,600 ^*b*^	2400
Respirable fused silica dust	560	13,600 ^*b*^	100

^*a*^ According to ref. [100], more than $5\times 10^{11}$ ultrafine particles per m^3^ were measured. Assuming them to be spheres with a diameter of 100 nm, and a density of 2.15 g/cm^3^ (corresponding to fused silica), this gives a concentration of more than 560 μg m^−3^. See also ref. [163] for discussion of these measurements, and [164,165] for further discussion of what constitutes a respirable particle. The corresponding WEL for respirable silicon, aluminum and aluminum oxide is slightly higher (4000 μg m^−3^). ^*b*^ Assumed to increase *pari passu* with the increase in T*o*CP.

## Data Availability

No new data were created or analyzed in this study.
